# Involvement of *FoxO1* in the effects of follicle-stimulating hormone on inhibition of apoptosis in mouse granulosa cells

**DOI:** 10.1038/cddis.2014.400

**Published:** 2014-10-16

**Authors:** M Shen, Z Liu, B Li, Y Teng, J Zhang, Y Tang, S-C Sun, H Liu

**Affiliations:** 1 Department of Animal Genetics, Breeding and Reproduction, College of Animal Science and Technology, Nanjing Agricultural University, Nanjing, China; 2 Institute of Animal Science, Chinese Academy of Agricultural Science, Beijing, China

## Abstract

In mammalian ovaries, follicular atresia occurs periodically and destroys almost all the follicles in the ovary. Follicle-stimulating hormone (FSH) acts as the primary survival factor during follicular atresia by preventing apoptosis in granulosa cells. FoxO1 is a critical factor in promoting follicular atresia and granulosa cell apoptosis. FSH inhibits the induction of *FoxO1*. In this report, we investigated the role of FSH-FoxO1 pathway in mouse follicular atresia. FSH dampened stress-induced apoptosis and the expression of *FoxO1* and pro-apoptosis genes in mouse granulosa cells (MGCs). In contrast, overexpression of FoxO1 inhibited the viability of MGCs and induced the expression of endogenous FoxO1. The signaling cascades involved in regulating FoxO1 activity upon FSH treatment were identified using FSH signaling antagonists. Blocking protein kinase A (PKA), phosphatidylinositol-3 kinase (PI3K) or protein kinase B (AKT) restored the upregulation of *FoxO1* and apoptotic signals, which was suppressed by FSH. Moreover, inhibition of PKA or PI3K impaired FSH-induced AKT activity, but inactivation of PI3K or AKT had little effect on PKA activity in the presence of FSH. Correspondingly, constitutive activation of FoxO1 (all three AKT sites were replaced by alanines) also promoted MGC apoptosis despite FSH administration. Furthermore, both luciferase reporter assays and chromatin immunoprecipitation assays showed that FoxO1 directly bound to a FoxO-recognized element site within the *FoxO1* promoter and contributed to the regulation of *FoxO1* expression in response to FSH. Taken together, we propose a novel model in which FSH downregulates FoxO1-dependent apoptosis in MGCs by coordinating the PKA–PI3K–AKT–FoxO1 axis and FoxO1–FoxO1 positive feedback.

More than 99% of mammalian ovarian follicles undergo degeneration during growth and development, a phenomenon known as follicular atresia.^1^ Inappropriate follicular atresia is responsible for certain reproductive disorders, such as polycystic ovarian syndrome and premature ovarian failure (also known as premature menopause), leading to infertility in women.^2,3^ Previous studies have demonstrated a close relationship between follicular atresia and granulosa cell apoptosis in which DNA fragmentation, activation of caspases and upregulation of pro-apoptotic gene expression are seen.^4^ Correspondingly, the maturation of follicles is a complex process that is regulated by gonadotropins and intraovarian regulators.^5,6^ In particular, follicle-stimulating hormone (FSH) is required for the production of estrogen,^7^ growth and development of antral follicles^8^ and the selection of dominant follicles (DFs).^9^ These physiological responses to FSH are achieved by activating several signaling cascades in granulosa cells, including protein kinase A (PKA), protein kinase B (PKB/AKT), p38 mitogen-activated protein kinase (p38-MAPK) and extracellular signal-regulated kinases 1 and 2 (ERK1/2), which in turn modulate >100 different target genes.^10^ The effect of FSH is due to its binding to FSH receptor, which is specifically localized on the plasma membrane of granulosa cells.^11^ FSH was identified as a major survival factor for antral follicles because of its ability to antagonize apoptosis in granulosa cells.^12^ However, its target genes and the potential mechanism for protection of granulosa cells during this stage remain to be elucidated.^13^

The FoxO subfamily of forkhead transcription factors, which includes FoxO1, FoxO3, FoxO4 and FoxO6, regulates genes required for apoptosis, cell cycle arrest, muscle regeneration, mitophagy, cellular homeostasis, aging and mitochondrial metabolism.^14^ FoxO activity is regulated by numerous post-translational modifications. When not phosphorylated, FoxO functions as a transcriptional activator or repressor by binding to the FoxO-recognized element (FRE) within the promoters of its target genes. Phosphorylation of FoxO by PKB/AKT in response to insulin, growth factors, hormones and other stimuli results in the exclusion of FoxO from the nucleus and subsequent degradation in the cytosol, inhibiting FoxO-dependent transcription.^15^ In the absence of insulin and/or growth factors, PKB/AKT suppression induces dephosphorylation and nuclear localization of FoxO, leading to cell cycle arrest and apoptosis via the activation of genes, such as cyclin-dependent kinase inhibitor (*p27KIP1*), BCL2-like 11 (*Bim*), tumor necrosis factor-related apoptosis-inducing ligand (*TRAIL*) and Fas ligand (*FasL*).^16, 17, 18, 19, 20^ Moreover, acetylation and mono-ubiquitination are other ways that FoxO activity is controlled.^21^

A recent study in the ovary suggests a potential function for FoxO1 in follicular development.^22^ An immunohistochemical assay demonstrated the centralization and nuclear distribution of FoxO1 staining in rat granulosa cells from atretic follicles.^23^ FoxO1 inhibits proliferation and steroidogenesis in mouse granulosa cells (MGCs), which may further induce apoptosis and follicular atresia. Conversely, FSH upregulates the expression of genes involved in cell proliferation and estrogen production, promotes follicular growth, and decreases granulosa cell apoptosis.^24^ The opposing activities of FoxO1 and FSH on follicular development and granulosa cell survival imply a reciprocal inhibition between these two factors in the ovary. As a target of FSH signaling, FoxO1 activity is negatively influenced by FSH.^22^ In granulosa cells, FSH induces phosphorylation of FoxO1 by PKB/AKT at several residues, resulting in retrotranslocation of FoxO1 from the nucleus to the cytoplasm.^25^ Although FoxO1 is abundant in cultured granulosa cells, FSH treatment significantly decreases its expression.^22,24,26,27^ Thus, FSH may mediate ovarian function in a FoxO1-dependent manner.^23^ However, few further clues exist regarding the regulation of FoxO1 in response to FSH signaling.

Our previous work demonstrated a critical role for FoxO1 in driving MGC apoptosis and follicular atresia.^4^ FSH has the opposite affect by facilitating follicular maturation and granulosa cell proliferation.^10^ Therefore, we were interested in the contribution of FoxO1 to FSH signaling cascades. As expected, we found that FSH significantly reduced *FoxO1* expression in MGCs both *in vivo* and *in vitro* in agreement with previous reports.^22,24,26,27^ Therefore, we hypothesized that downregulation of FoxO1-induced apoptosis may correlate with the action of FSH on granulosa cell survival. In this study, we investigated the response mechanism of FoxO1 to FSH-mediated prevention of apoptosis in MGCs. Our results suggested a primary role for FoxO1 inhibition of FSH-induced MGC survival through coordination of the PKA–phosphatidylinositol-3 kinase (PI3K)–AKT–FoxO1 axis and FoxO1–FoxO1 positive feedback.

## Results

### FSH protected MGCs from apoptosis in dominant ovarian follicles

**Figure 1:**
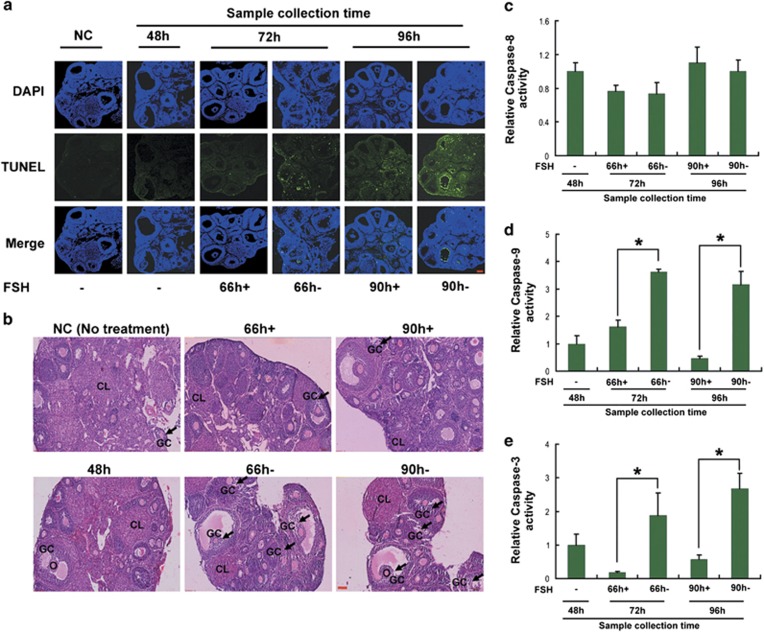
FSH reduced apoptosis in MGCs of ovarian DFs. Mice were injected i.p. with FSH twice daily (12-h intervals, 08 00 and 2000 hours) for 2 days at a dose of 10 IU on day 1 and 5 IU on day 2. FSH was then withdrawn for an additional 24 or 48 h, or injected i.p. (10 IU per mouse) 6 h before MGC collection. (**a**–**e**) At 48, 72 and 96 h after the first FSH injection, the right ovaries were paraffin-embedded and serially sectioned for the TUNEL assay (**a**) and H&E staining (**b**). MGCs were isolated from DFs (>200 *μ*m) in the left ovaries for caspase activity detection (**c**–**e**). The data represent the mean±S.E. (*n*=3). *P<0.05, Student's *t*-test. Bar, 100 *μ*m. O, oocyte; GC, granulosa cells; CL, corpus luteum

It is well established that FSH is the primary survival factor for DFs.^28^ FSH alone promotes antral follicles growth and development into preovulatory follicles, which will maintain anovulation without the stimulation of leutinizing hormone (LH).^29^ FSH withdraw (coasting) during this stage leads to granulosa cell apoptosis and follicular atresia.^30^ We therefore developed a corresponding FSH treatment protocol to mimic *in vivo* DFs growth and atresia as shown in Materials and Methods section and Supplementary Figure S1. In brief, the growth of mouse ovarian DFs was induced by intraperitoneal (i.p.) injection with FSH twice daily (12-h intervals) for 2 days at a dose of 10 IU on day 1 and 5 IU on day 2. FSH was then withdrawn for an additional 24 or 48 h to stimulate physiological follicular atresia in DFs, or injected i.p. (10 IU per mouse) 6 h before MGC retrieval. At 48, 72 and 96 h after the first FSH injection, we collected mouse ovaries or MGCs of DFs for tests. Using the terminal deoxynucleotidyl transferase-mediated dUTP nick end labeling (TUNEL) assay, MGC apoptosis was significantly increased after 24 and 48 h of FSH deprivation (66 and 90-h groups). Specifically, TUNEL-positive staining was concentrated in MGCs within DFs. In contrast, mice primed with FSH 6 h before FSH withdrawal showed low apoptotic signals in ovarian MGCs (Figure 1a). Using hematoxylin and eosin (H&E) staining, we detected the effects of FSH on follicular atresia under the same conditions. Consistent with the data from the TUNEL assay, mice in which FSH was withdrawn displayed more atretic follicles (including DFs) in ovaries compared with those supplemented with FSH (Figure 1b).

A previous study suggested the involvement of a mitochondria-dependent pathway in initiating granulosa cell apoptosis.^31^ To investigate the correlation of this pathway with FSH-induced granulosa cell protection, we analyzed the activities of caspases in MGCs and found that upregulation of caspase-3 and caspase-9 (mediates mitochondria-dependent apoptosis) activity was blocked by additional injection of FSH during the FSH withdrawal (Figures 1d and e). However, no significant change in caspase-8 activity (responsible for mitochondria-independent apoptosis) was observed in response to FSH treatment (Figure 1c). Consistent with this, we showed that the mRNA expression of *Bim*, but not *FasL*, is regulated by FSH.^32^ These results showed that FSH may prevent ovarian MGC apoptosis by inhibiting the mitochondria-dependent pathway.

### FSH reduced FoxO1 expression in ovarian MGCs

**Figure 2:**
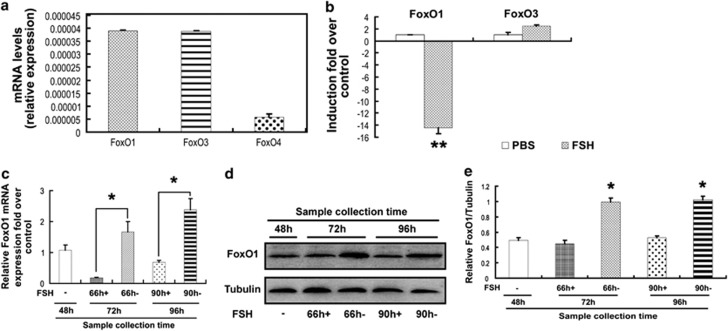
FSH protected follicular MGCs from apoptosis in a FoxO1-dependent manner. Ovarian stimulation and MGC retrieval were performed as described in Supplementary Figure S1. (**a**) The expression of FoxO family members in MGCs of DFs was measured with qRT-PCR. (**b**) The responsiveness of *FoxO1* and *FoxO3a* to FSH treatment was detected as above. (**c**) Transcription changes in *FoxO1* in response to FSH-induced MGC protection. (**d**) The inhibition of FoxO1 expression by FSH was examined using western blotting. *α*-Tubulin served as the loading control. (**e**) Quantification of relative FoxO1 protein levels with densitometry. ImageJ 1.42q software was used to analyze the density of each band in Figure 2**d**, and the relative expression level was normalized to that of *α*-tubulin. The data represent the mean±S.E. (*n*=3). *P<0.05; **P<0.01, Student's *t*-test

The effects of FSH on FoxO1 expression were measured with quantitative reverse transcription polymerase chain reaction (qRT-PCR) and western blotting. We first examined the mRNA levels of *FoxO* family members in MGCs collected from ovarian DFs 96 h after the original FSH injection in mice and found that *FoxO1* and *FoxO3a* were the most abundant *FoxO* mRNA isoforms in MGCs of DFs (Figure 2a), in agreement with previous observations.^33^ We next evaluated the responsiveness of *FoxO1* and *FoxO3a* to FSH treatment. *FoxO1* mRNA was inhibited by approximately 14-fold by FSH administration during the FSH withdrawal. However, only about a one- to twofold increase in *FoxO3a* mRNA occurred under the same conditions (Figure 2b). These data suggested that FSH specifically regulated *FoxO1* rather than other *FoxO* mRNAs in MGCs of DFs, and we thus focused our subsequent studies on *FoxO1*. qRT-PCR analysis indicated that the high level of *FoxO1* mRNA in MGCs induced by FSH deprivation was significantly decreased in mice primed with FSH 6 h before FSH deprivation (Figure 2c). Consistent with this observation, FSH treatment suppressed the expression of FoxO1 protein in MGCs retrieved from DFs during FSH withdrawal (Figures 2d and e). The synchronization of MGC protection and FoxO1 inhibition by FSH reflects a potential connection between FoxO1 and FSH. In addition, earlier studies had identified FoxO1 as a transactivator of Bim,^15,18^ which is a component of the mitochondria-dependent apoptotic pathway^34^ that is also downregulated by FSH as mentioned above. Therefore, our data raised the possibility that FSH prevents MGCs from undergoing apoptosis in a FoxO1-dependent manner.

### FSH protected MGCs from apoptosis *in vitro*

**Figure 3:**
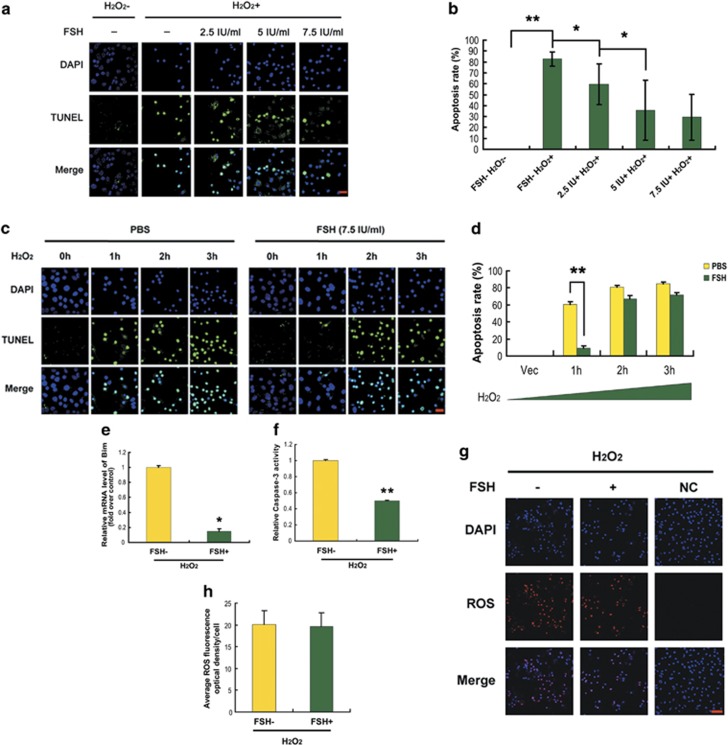
FSH suppressed apoptosis in cultured MGCs. (**a**) Granulosa cells collected from DFs were cultured in serum-free medium containing 200 *μ*M H_2_O_2_ and FSH at different concentrations for 6 h. The apoptotic signals were visualized with TUNEL staining (green), and the nuclei were counterstained with DAPI (blue). Bar, 20 *μ*m. (**b**) Quantification of the apoptosis rates by calculating the average number of TUNEL-positive nuclei per visual field. Experiments were repeated three times, and cells were counted in three randomly selected fields from each coverslip. Data represent the means±S.E. The statistical significance between groups was analyzed with one-way ANOVA, and bars labeled with different letters differ significantly at *P*<0.01 (**a** and **b**) or *P*<0.05 (**a** and **b**). (**c**) After incubation with 200 *μ*M H_2_O_2_ for 0 (vehicle), 1, 2 or 3 h, MGCs were rinsed with PBS and cultured in serum-free medium containing 7.5 IU/ml FSH for 12 h. Apoptosis was detected as above. Bar, 20 *μ*m. (**d**) Quantification of apoptosis rates in each treatment as shown in (**c**). Data represent the means±S.E. (**e**–**g**) MGCs were incubated for 1 h in 200 *μ*M H_2_O_2_, washed with PBS, plated in serum-free medium with 7.5 IU/ml FSH for 12 h, and then processed for qRT-PCR for *Bim* (**e**), caspase-3 activity assay (**f**) and intracellular ROS detection (**g**). Bar, 50 *μ*m. (**h**) Quantification of intracellular ROS levels. The optical density was evaluated in each MGC with ImageJ 1.42q software. Data represent mean±S.E. (*n*=3). *P<0.05; **P<0.01, Student's *t*-test

Follicular growth and granulosa cell survival are controlled by both ovarian steroids and pituitary gonadotropins.^35^ To further confirm the anti-apoptotic potential of FSH and to rule out the possible actions of other endocrine factors, experiments were performed with cultured granulosa cells harvested from DFs. Exposure to H_2_O_2_ (200 *μ*M) induced apoptotic death in about 85% of MGCs 6 h later, and this death was remarkably suppressed by FSH in a dose-dependent manner (Figures 3a and b). FSH at 7.5 IU/ml tended to show maximal protection and thus was chosen for subsequent experiments. We next studied the time course of the viability of MGCs upon H_2_O_2_ treatment in the presence of FSH. Cells incubated in 200 *μ*M H_2_O_2_ for 0, 1, 2 or 3 h were washed with phosphate-buffered saline (PBS) and then cultured in serum-free medium containing 7.5 IU/ml FSH for 12 h. Apoptotic signals were indistinguishable from cells with 0 h of H_2_O_2_ exposure. H_2_O_2_ incubation for 1 h led to a marked increase in the rate of TUNEL-positive cells, which was significantly blocked by FSH. However, apoptosis triggered by >2 h of H_2_O_2_ exposure could not be rescued by FSH (Figures 3c and d and Supplementary Figure S2). Therefore, 1 h of H_2_O_2_ treatment was considered a ‘rescueable' length of time to induce apoptosis in MGCs and was applied in the following experiments. In accordance with the TUNEL assay, qRT-PCR and caspase-3 activity analysis showed that induction of the pro-apoptotic genes, Bim and caspase-3, by H_2_O_2_ was significantly decreased by FSH (Figures 3e and f) in cultured MGCs. These observations were consistent with the results of *in vivo* experiments. To determine whether an indirect effect of FSH may influence MGC survival, we asked if FSH inhibited apoptosis by reducing oxidative stress. Our data showed no significant change in reactive oxygen species (ROS) levels following FSH treatment (Figures 3g and h), suggesting a direct action of FSH on MGC protection. These results indicated that FSH decreased apoptosis in cultured MGCs.

### FSH attenuated FoxO1 expression in cultured MGCs

**Figure 4:**
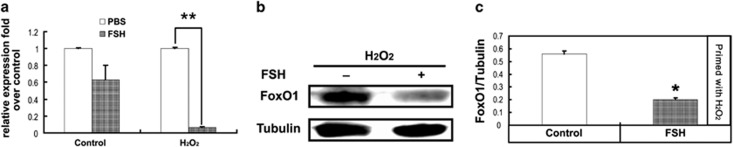
FoxO1 expression was downregulated by FSH *in vitro*. Primary MGCs collected from DFs were exposed to 200 *μ*M H_2_O_2_ for 1 h, rinsed with PBS and cultured in serum-free medium containing 7.5 IU/ml FSH for 12 h. (**a**) The effects of FSH on *FoxO1* expression in MGCs were determined with qRT-PCR. Expression data were normalized to that of *β*-actin. (**b**) Western blotting analysis of FoxO1 protein levels in cultured MGCs treated with or without FSH. *α*-Tubulin was used as the loading control. (**c**) Quantification of the relative FoxO1 protein level with densitometry. ImageJ 1.42q software was used to analyze the density of each band in Figure 4**b**, and the relative expression level was normalized to that of *α*-tubulin. The data represent the mean±S.E. (*n*=3). *P<0.05; **P<0.01, Student's *t*-test

Few reports have reported follicle stage-specific regulation of FoxO1 by FSH in granulosa cells. In this study, we determined whether *FoxO1* expression was correlated with FSH in MGCs from DFs. FSH blocked the induction of *FoxO1* in MGCs of DFs during the FSH withdrawal (Figures 2c–e). To validate the *in vivo* results, *FoxO1* expression levels were examined in cultured MGCs isolated from ovarian DFs. As shown in Figure 4a, cells pre-treated with H_2_O_2_ displayed a marked decrease in *FoxO1* mRNA (14.5-fold) following FSH incubation compared with the control group (1.6-fold), consistent with the observations from the TUNEL assay under the same conditions (Figures 3c and d). Western blotting results also confirmed the inhibition of FoxO1 protein levels by FSH (Figures 4b and c). Taken together, the evidence from *in vivo* and *in vitro* experiments demonstrated that FSH repressed FoxO1-dependent apoptosis in MGCs of DFs.

### FSH promoted MGC survival through the PKA–PI3K–AKT–FoxO1 pathway

**Figure 5:**
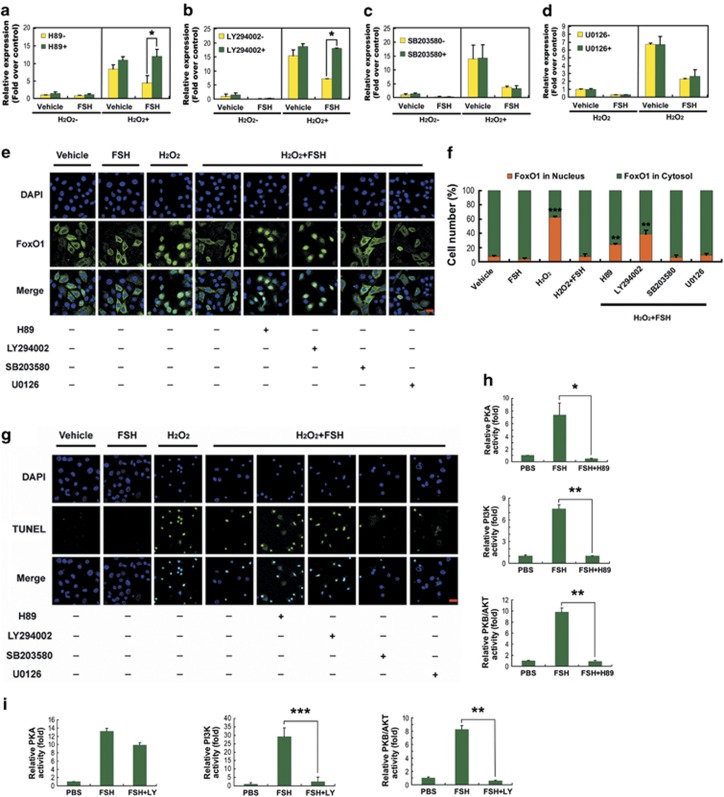
FSH prevented MGCs from undergoing apoptosis *via* the PKA–PI3K–AKT–FoxO1 axis. Primary cultured MGCs were exposed to 200 *μ*M H_2_O_2_ for 1 h and then grown in serum-free medium containing 7.5 IU/ml FSH for 2 h (in **e**, **f**, **h** and **i**) or 6 h (in **a**–**d** and **g**). H89 (10 *μ*M), LY294002 (20 *μ*M), SB203580 (20 *μ*M) or U0126 (3 *μ*M) was added for 30 min before FSH treatment. (**a**–**d**) The effects of the FSH signaling antagonists on *FoxO1* expression were determined with qRT-PCR. Expression data were normalized to that of *β-actin*. (**e**) Subcellular localization of FoxO1 in response to the indicated FSH signaling inhibitors was detected using anti-FoxO1 (green), and the nuclei were counterstained with DAPI (blue). Bar, 20 *μ*m. (**f**) The percentage of cells with FoxO1 in the nucleus (orange bars) and in the cytosol (green bars). Experiments were performed in triplicate, and cells were counted in three randomly selected fields in each coverslip. Data represent the mean±S.E. (**g**) The effects of the FSH signaling antagonists on stress-induced apoptosis in cultured MGCs. The apoptotic signals were visualized with TUNEL staining (green), and the nuclei were counterstained with DAPI (blue). Bar, 20 *μ*m. (**h**) The effects of the PKA inhibitor H89 on the activity of PKA, PI3K and AKT. (**i**) The effects of the PI3K inhibitor LY294002 on the activity of PKA, PI3K and AKT. The data represent the mean±S.E. (*n*=3). *P<0.05; **P<0.01; ***P<0.001, Student's *t*-test

FSH activates multiple downstream signaling cascades in granulosa cells, including PKA, PI3K, PKB/AKT, p38-MAPK and ERK1/2.^10^ To further investigate the mechanisms of *FoxO1* regulation by FSH, we explored the role of these pathways in the inhibition of *FoxO1* and apoptosis in primary cultured MGCs retrieved from DFs. qRT-PCR analysis showed that H89 (PKA inhibitor) and LY294002 (PI3K inhibitor) abolished the downregulation of *FoxO1* expression by FSH (Figures 5a and b), whereas SB203580 (p38-MAPK inhibitor) and U0126 (ERK1/2 inhibitor) had no effect (Figures 5c and d). Considering the role of PI3K/AKT in stimulating the cytoplasmic distribution and inactivation of FoxO1,^15^ we thus evaluated the impact of these pathways on FoxO1 subcellular localization. Immunofluorescence staining indicated that the actions of FSH in suppressing H_2_O_2_-derived nuclear translocation of FoxO1 were reversed by H89 and LY294002, but not SB203580 or U0126 (Figures 5e and f). Consistent with this, compared with SB203580 and U0126, treatment with both H89 and LY294002 restored the H_2_O_2_-triggered apoptosis, which was significantly decreased by FSH (Figure 5g). These data suggested a possible modulation by FSH of FoxO1-dependent apoptosis in MGCs through the PKA and PI3K/AKT pathways. We then examined the relationship among PKA, PI3K and AKT. As shown in Figures 5h and i, all three kinases were induced by FSH treatment. Blocking PKA or PI3K reduced AKT activity, but inactivation of PI3K and/or AKT had little effect on PKA activity. In contrast, the PKA inhibitor blunted the induction of both PI3K and AKT. Therefore, the PKA–PI3K–AKT–FoxO1 pathway may be involved in FSH-mediated MGC protection.

### Constitutively active FoxO1 in MGCs negated the protection from FSH

**Figure 6:**
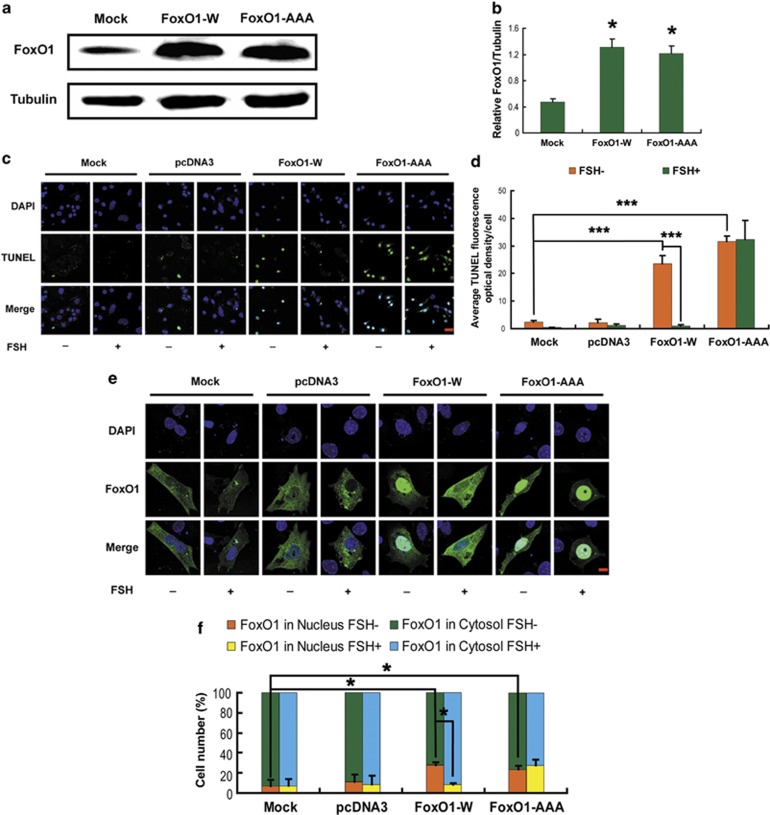
Activation of FoxO1 in MGCs abolished the anti-apoptotic effects of FSH. Primary MGCs were transfected with WT FoxO1 (FoxO1-W), a constitutively active FoxO1 mutant (FoxO1-AAA, all three AKT sites were replaced by alanines) or a control vector (pcDNA3) for 36 h. (**a**) Western blotting analysis of FoxO1 protein levels in cultured MGCs transfected with FoxO1 expression vectors. *α*-Tubulin was used as the loading control. (**b**) Quantification of relative FoxO1 protein levels with densitometry. ImageJ 1.42q software was used to analyze the density of each band, and the relative expression level was normalized to that of *α*-tubulin. (**c**) Cells were treated with or without FSH (7.5 IU/ml) during transfection. The apoptotic signals were detected with FITC fluorescence (green), and the nuclei were counterstained with DAPI (blue). Bar, 20 *μ*m. (**d**) Quantification of the apoptotic signals. The optical density was evaluated in each MGC with ImageJ 1.42q software. (**e**) Cells were incubated with or without FSH (7.5 IU/ml) before collection. Subcellular localization of FoxO1 upon FSH stimulation was visualized using anti-FoxO1 (green), and the nuclei were counterstained with DAPI (blue). Bar, 10 *μ*m. (**f**) The percentage of cells with FoxO1 in the nucleus or the cytosol under the indicated conditions. Experiments were performed in triplicate, and cells were counted in three randomly selected fields on each coverslip. *P<0.05; ***P<0.001, Student's *t*-test

Our data revealed the FSH signaling that is required for downregulation of FoxO1 expression and apoptosis in MGCs of DFs. Our results in Figures 5e and f also suggested the influence of FSH on FoxO1 activity through PKA–PI3K–AKT. To further confirm the role that FoxO1 activity had in this pathway, we overexpressed wild-type (WT) FoxO1 (FoxO1-W) and a constitutively active FoxO1 mutant (FoxO1-AAA, the three AKT phosphorylation sites of FoxO1 are mutated to alanine) in cultured MGCs collected from DFs. Western blot analysis demonstrated a significant increase in FoxO1 expression after transfection. Equal amounts of FoxO1 protein levels were observed in both the FoxO1-W and FoxO1-AAA groups (Figures 6a and b), enabling us to specify the effects of FoxO1 activity without differences in FoxO1 expression. According to the TUNEL assay, both FoxO1 plasmids induced intense apoptotic signals in MGCs that were blocked by FSH in the FoxO1-W group. Conversely, expression of FoxO1-AAA abolished FSH-induced cell survival (Figures 6c and d). In accordance with this, immunofluorescence staining indicated that FSH inhibited overexpression-triggered nuclear translocation of FoxO1 in the FoxO1-W group, but not in the FoxO1-AAA group (Figures 6e and f). These observations suggested the importance of FoxO1 activity in the response to the FSH-activated PKA–PI3K–AKT axis in MGCs.

### The self-reinforcing regulation of FoxO1 in MGCs

**Figure 7:**
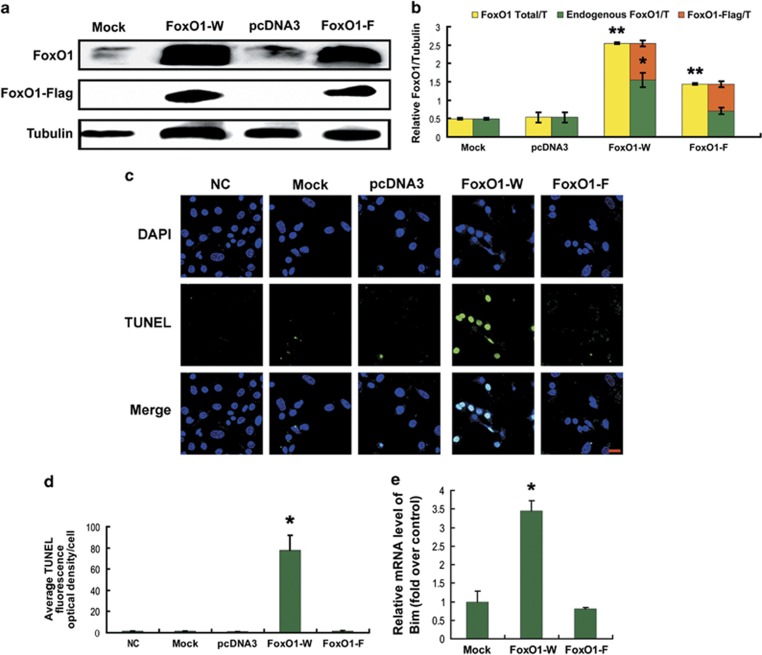
FoxO1-induced apoptosis in MGCs by activating the expression of FoxO1 and downstream apoptotic genes. Primary cultured MGCs harvested from DFs were transfected with pcDNA3-FLAG-FKHR (FoxO1-W), pcDNA3-FLAG-FKHR;H215R (FoxO1-F, containing a point mutation in the DBD), or a control vector (pcDNA3) for 36 h. (**a**) Western blotting analysis of FoxO1 protein levels. *α*-Tubulin was used as the loading control. (**b**) Quantification of relative FoxO1 protein levels with densitometry. ImageJ 1.42q software was used to analyze the density of each band, and the relative expression level was normalized to that of *α*-tubulin. Yellow bar, total FoxO1 protein. Green bar, endogenous FoxO1 protein. Orange bar, exogenous FoxO1 protein. (**c**) The apoptotic signals in MGCs after transfection were detected with FITC fluorescence (green), and the nuclei were counterstained with DAPI (blue). Bar, 20 *μ*m. (**d**) Quantification of the apoptotic signals. The optical density was evaluated in each MGC with ImageJ 1.42q software. Experiments were performed in triplicate, and cells were counted in three randomly selected fields in each coverslip. Data represent the means±S.E. (**e**) Transcription changes in *Bim* in response to FoxO1 overexpression-induced apoptosis in MGCs. Expression data were normalized to that of *β*-actin. *P<0.05; **P<0.01, Student's *t*-test

As both FoxO1 expression and activity participated in FSH-initiated apoptotic control in MGCs through the PKA–PI3K–AKT axis, we next sought to examine their connection in this process. FoxO3a was previously reported to bind to the *FoxO1* promoter region and control FoxO1 expression.^36, 37, 38^ As all FoxO family members have a consensus DNA-binding sequence known as FRE,^15^ we hypothesized that FoxO1 triggered apoptosis by upregulating FoxO1 expression in MGCs of DFs when FSH signaling was absent. To test this hypothesis, MGCs retrieved from DFs were transfected with pcDNA3-FLAG-FKHR (W) or pcDNA3-FLAG-FKHR;H215R (F, containing a point mutation in the DNA-binding domain (DBD)). As shown in the western blot, overexpression of WT FoxO1 significantly increased endogenous FoxO1 expression, whereas the DBD mutant had no effect (Figures 7a and b). In accordance with this, the DBD mutant lost the activator effect of FoxO1 overexpression on apoptosis in MGCs (Figures 7c and d). In addition, considering that FRE sites are also found in the *Bim* promoter,^18^ we measured *Bim* expression with qRT-PCR under the same conditions. Compared with MGCs transfected with WT FoxO1, DBD mutation had little effect on the induction of intracellular mRNA levels of Bim (Figure 7e). Collectively, our data indicated that FoxO1 enhanced the expression of FoxO1 and its downstream apoptotic gene, thus leading to apoptosis in MGCs.

**Figure 8:**
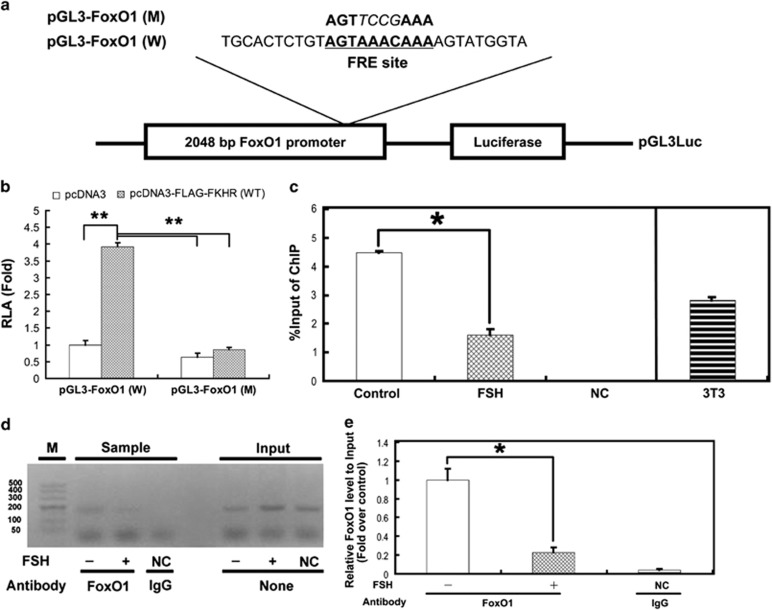
FoxO1 directly bound to the FRE site in the *FoxO1* promoter. (**a**) A 2048-bp fragment of the *FoxO1* promoter was amplified with PCR using mouse genomic DNA (bases 78–2125, 5'-UTR of *FoxO1*) containing a FRE (AGTAAACAAA) and cloned into the pGL3-Basic plasmid as pGL3-FoxO1 (W). pGL3-FoxO1 (M) was obtained by introducing four mutations (italics) into the FRE site. (**b**) *FoxO1* reporter activities in mouse 3T3 cells co-transfected with FoxO1 expression vectors and *FoxO1* promoter constructs for 36 h. The reporter activities were normalized to those of pRL-TK. RLA, relative luciferase activities. (**c**–**e**) The binding of FoxO1 to the *FoxO1* promoter in MGCs or 3T3 fibroblasts was detected with ChIP assays following FSH treatment for 6 h. DNA was isolated from the precipitated complexes as a template for qRT-PCR (**c**). The qRT-PCR products were then analyzed on a 2% agarose gel (**d**) and quantified with densitometry using ImageJ 1.42q software (**e**). The amount of immunoprecipitated DNA for each ChIP reaction is shown as the percentage of input chromatin. IgG was the negative control. The data represent the means±S.E. (*n*=3). *P<0.05; **P<0.01, Student's *t*-test

To further test if FoxO1 directly activated the expression of its own gene at the promoter level, a 2048-bp fragment of the *FoxO1* 5' UTR containing the putative FRE site was cloned in front of a luciferase reporter gene as pGL3-FoxO1 (W) (Figure 8a and Supplementary Figure S3). This construct was then co-transfected with pRL-TK and pcDNA3-FLAG-FKHR (W) or the control pcDNA3 into NIH 3T3 fibroblasts. WT FoxO1 but not the pcDNA3 vector markedly heightened FoxO1 promoter activity. In contrast, FRE site mutation significantly decreased the responsiveness of the reporter to FoxO1 overexpression (Figure 8b). We next performed chromatin immunoprecipitation (ChIP) experiments to investigate the binding of FoxO1 to the *FoxO1* promoter. MGC and NIH 3T3 fibroblast chromatin was first sheared into fragments of 250–750 bp with sonication (Supplementary Figure S6) and then used for immunoprecipitation. As shown in Figures 8c–e, the anti-FoxO1 antibody precipitated the *FoxO1* promoter region containing the FRE site in chromatin from both MGCs and NIH 3T3 fibroblasts. Importantly, the signals in MGCs were markedly inhibited following FSH treatment for 6 h. Together, these results suggested that FoxO1 was directly recruited to the FRE site in the *FoxO1* promoter for transcriptional activation in the absence of FSH.

Moreover, we asked if FSH signaling affected *FoxO1* expression via other transcriptional factors. We first predicted the transcriptional factor binding sites in the *FoxO1* promoter using TFSEARCH, Promoter Scan and GeneCards and selected candidate genes for qRT-PCR analysis. Of the six genes with detectable mRNA levels in MGCs, a strong correlation between the expression patterns of estradiol receptor (ER) alpha and *FoxO1* (*P*<0.01) in response to FSH and PKA inhibitor treatment was observed (Supplementary Figure S4), indicating that ERalpha may also be required for *FoxO1* induction upon FSH deprivation.

In conclusion, our data demonstrated that the PKA–PI3K–AKT–FoxO1 axis and FoxO1–FoxO1 positive feedback were important mechanisms for FSH-mediated MGC survival in ovarian DFs.

## Discussion

The regulation of follicular atresia requires complex interactions between gonadotropins (FSH and LH) and ovarian regulatory factors^39^ for determination of granulosa cell fate (proliferation, differentiation or programmed cell death).^6^ Interestingly, as a transcription factor responsible for cell growth arrest and apoptosis in multiple types of mammalian cells,^40^ FoxO1 is selectively and highly expressed in granulosa cells of mammalian growing follicles.^39^ In MGCs, FoxO1 activity is controlled by steroid hormones,^39^ whereas overexpression of FoxO1 reduces steroidogenesis.^22^ In addition, immunohistochemistry showed intense nuclear staining of FoxO1 that was centralized in rat granulosa cells from atretic follicles.^23^ Our previous work also identified FoxO1 as a critical factor in oxidative stress-triggered MGC apoptosis.^4^ These reports suggested a possible contribution of FoxO1 to follicular atresia.^22,25^ Nevertheless, no direct evidence has been obtained indicating that FoxO1 performs the same role under physiological conditions. Gonadotrophins (FSH and LH) are primarily responsible for the selection of DFs.^41^ FSH alone promotes antral follicle growth and development into preovulatory follicles, which will maintain anovulation without stimulation by LH.^29^ FSH withdrawal (coasting) during this stage leads to granulosa cell apoptosis and follicular atresia.^30^ We therefore developed a corresponding FSH treatment protocol to mimic *in vivo* growth and atresia of DFs (Materials and Methods section and Supplementary Figure S1). According to our treatment procedure, we observed a clear association between MGC apoptosis and *FoxO1* expression in DFs (Figures 1 and 2), providing new evidence for the pro-apoptotic effect of FoxO1 in MGCs.

Previous studies indicated that the actions of FSH on ovarian functions are partially mediated by FoxO1.^23,39^ For example, the FSH-induced expression of genes required for lipid, sterol and steroid synthesis in MGCs is attenuated by a constitutively active FoxO1 mutant.^22^ In contrast, FSH blocks the inhibition of granulosa cell proliferation and estrogen production from FoxO1.^22,24^ Further research in granulosa cells showed that FoxO1 represses transcription of cyclin D2, thereby blocking cell cycle progression at the G0 phase. Also, FoxO1 prevents granulosa cell differentiation by downregulating steroidogenic factor-1, inhibin-*α*, aromatase cytochrome P-450 and epiregulin.^25^ Therefore, FSH-mediated *FoxO1* suppression will promote granulosa cell proliferation and differentiation.^10^ However, whether FoxO1 is correlated with the effects of FSH on antagonizing granulosa cell apoptosis remains to be determined. In this study, we demonstrated that FSH significantly decreased granulosa cell apoptosis and/or follicular atresia in the mouse ovary (Figures 1 and 3), consistent with previous reports.^32,42, 43, 44^ Importantly, MGC apoptosis that was reduced by FSH treatment was associated with downregulation of *FoxO1* expression (Figures 2 and 4). Thus, FSH may impair apoptosis in MGCs in a FoxO1-dependent manner.

Mammalian ovaries contain follicles in various stages of development.^9^ Although *FoxO1* is a target of FSH signaling,^25,39,45^ few studies have been performed on the follicle stage-specific modulation of *FoxO1* by FSH. Studies from others demonstrated that FSH treatment significantly decreases *FoxO1* levels in cultured granulosa cells. However, exposure of developing follicles to FSH enhances the expression of *FoxO1*, which is downregulated as granulosa cells differentiate to luteal cells.^22,24,26,27,39^ These contradictory observations suggested that FSH has distinct effects on *FoxO1* expression in granulosa cells at different follicular stages, and thus, we explored the correlation between *FoxO1* and FSH in MGCs from DFs. Our data indicated a synchronization of protection of MGCs and/or DFs and *FoxO1* inhibition that was mediated by FSH (Figures 1–4), supporting the concept that FSH is the major survival factor for DFs.^28^

Two major cascades lead to apoptosis: the extrinsic pathway (type I cell death pathway), which activates cell surface receptors in response to external signals such as FasL, and the intrinsic pathway (type II cell death pathway), which causes cytochrome c release into the cytosol following mitochondrial membrane disruption that is induced by Bcl-2 family members.^46^ Previous studies identified Bim as a BH3-only protein that promotes apoptosis by changing the balance between pro- and anti-apoptotic members of the Bcl-2 family, thereby affecting permeability of the mitochondrial membrane to cytochrome c, which further triggers the activation of caspase-9, caspase-3 and the cell death program.^47^ Initiation of apoptosis occurs in granulosa cells through the mitochondria-dependent pathway.^31^ As shown in this study (Figure 1) together with our previous results,^32^ MGC apoptosis driven by FSH withdrawal induced hallmarks of mitochondrial apoptosis (Bim, caspase-9 and caspase-3), which were then suppressed in the presence of FSH. In contrast, FSH had no effects on the components of death receptor signaling (FasL and caspase-8). These data suggested the involvement of the type II cell death pathway in FSH-mediated MGC protection. In addition, previous evidence indicated that FoxO1 regulates apoptosis by targeting several pro-apoptotic genes, including *Bim.*^18,19,48, 49, 50^ Our results showed that *Bim* followed a similar expression pattern as *FoxO1* during FSH treatment. Therefore, *Bim* appears to be a convergence point of the mitochondrial apoptotic pathway and FoxO1-dependent apoptosis that both contribute to FSH-induced MGC survival in DFs.

FSH regulates the growth and differentiation of follicular granulosa cells through several downstream signaling pathways, including PKA, PI3K, AKT, p38-MAPK and ERK1/2, although their interactions in response to FSH remain to be investigated.^10^ Previous studies indicated that FoxO1, the major downstream effector of PI3K/AKT, is phosphorylated following exposure to FSH in granulosa cells.^25,39,45^ In this study, it was found that both PKA and PI3K/AKT were activated by FSH in MGCs of DFs to inhibit the expression of *FoxO1* following stress-triggered apoptosis (Figures 5a and b). In contrast, H89 (PKA inhibitor) and LY294002 (PI3K inhibitor) diminished the protection against apoptosis provided by FSH in cultured MGCs that was associated with suppression of FoxO1 activity (Figures 5e–g). Correspondingly, overexpression of a constitutively active FoxO1 mutant (all three AKT sites were replaced by alanines) in MGCs abolished the actions of FSH on repressing *FoxO1* and apoptosis (Figure 6). Further experiments using FSH signaling antagonists identified PKA as an upstream activator of PI3K, which in turn stimulated the activation of AKT (Figures 5h and i). These findings were consistent with a role for PKA in initializing FSH-induced granulosa cell survival as described previously.^51, 52, 53^ MAPK-related proteins, such as JNK, ERK1/2 and p38-MAPK, regulate FoxO1 activity,^54^ stress resistance^55^ and FSH response.^56^ However, no definitive evidence was observed for the involvement of either ERK1/2 or p38-MAPK in preventing MGC apoptosis or *FoxO1* induction with FSH treatment (Figures 5c–g). Taken together, our results suggested that PKA–PI3K–AKT–FoxO1 may be a major axis for FSH-mediated MGC protection.

The potent functions of FoxO1 are tightly controlled by its transcription and phosphorylation states.^40,57^ Although it is well understood that phosphorylation of FoxO1 by PKB/AKT protects cells from apoptosis because of the nuclear exclusion of phospho-FoxO1 and thereby blocking transactivation of pro-apoptotic genes including *p27KIP1*, *Bim*, *TRAIL* and *FasL*,^16, 17, 18, 19, 20^ the interplay between phosphorylation and expression in the regulation of FoxO1 expression has not been entirely characterized, especially in granulosa cells.^39^ As mentioned above in this study, the inactivation of FoxO1 by PKA–PI3K–AKT may also be attributed to its phosphorylation and subsequent sequestration in the cytoplasm (Figures 5 and 6). However, mechanisms regarding the inhibition of FoxO1 expression through this signaling axis remain to be explored. Our results showed that overexpression of WT FoxO1 stimulated endogenous FoxO1 expression and *FoxO1* promoter activity, as well as elevation of apoptotic signals in MGCs (Figures 7 and 8). In fact, FoxO1 bound directly to the FRE site within the *FoxO1* promoter (Figure 8), whereas phosphorylation of FoxO1 by FSH dissociated FoxO1 from its DNA-binding sequence, which was correlated with the downregulation of *FoxO1* expression (Figures 2,4–6 and 8). Therefore, FSH-induced *FoxO1* suppression may also terminate the transcriptional induction of its downstream pro-apoptotic effectors such as *Bim* (Figures 3 and 7). These data suggested that when FSH signaling is absent, FoxO1 activates the transcription of *FoxO1* and its downstream apoptotic genes, thereby triggering apoptosis in MGCs. Consistent with our observations, earlier reports indicated the ability of FoxO3a to bind and activate both the *FoxO1* and *FoxO3a* promoters in other cell types.^36, 37, 38,58^ As all FoxO family members share the FRE consensus DNA-binding sequence,^15^ such positive feedback regulation may be a common mechanism among the *FoxO* genes. A recent study in porcine granulosa cells showed that FSH inhibits nuclear accumulation of FoxO3a by PI3K/AKT-mediated phosphorylation.^13^ Therefore, although FSH has little effect on the expression of *FoxO3a* (Figure 2b), whether FSH-regulated FoxO3a activity exerts any influence on *FoxO1* expression remains to be investigated in MGCs. Nevertheless, our study sheds new light on the connection between *FoxO1* expression and FoxO1 activity in FSH-mediated MGC survival. A hypothetical model (Supplementary Figure S5) shows FSH regulation of FoxO1-dependent apoptosis in MGCs of ovarian DFs.

Moreover, FSH stimulates granulosa cells to secrete estradiol, a steroid hormone that promotes ovarian follicle survival and growth via two ERs, ERalpha and ERbeta.^59^ In this study, a positive correlation between FoxO1 and ERalpha expression was seen in response to FSH and PKA inhibitor treatment both *in vitro* (Supplementary Figure S4) and *in vivo* (data not shown) in MGCs. Previous reports indicated that ERalpha and FoxO1 proteins are downstream targets of estradiol.^60^ Proteomic analysis showed the binding of ERalpha and FoxO1 on estrogen response elements.^61^ Similarly, FoxO1 colocalizes with ERalpha in the nucleus of breast cancer cells, but estradiol-induced FoxO1 phosphorylation led to nuclear export of the FoxO1-ERalpha complex.^62^ Although these findings demonstrated coregulation of ERalpha and FoxO1, our results suggest a relationship at the transcription level. However, further experiments such as ChIP, luciferase reporter assays, qRT-PCR, western blotting and TUNEL staining should be conducted to confirm the effects of ERalpha on FoxO1 expression during FSH-induced MGC survival.

In summary, this study describes a mechanism underlying FSH-mediated protection against apoptosis in MGCs by suppressing *FoxO1* through the PKA–PI3K–AKT–FoxO1 axis and FoxO1–FoxO1 positive feedback. Further identifying the functions of the upstream regulators and downstream effectors of *FoxO1* should provide novel insights into the exact regulation modes of *FoxO1*-dependent apoptosis upon FSH stimulation during follicular atresia. Therefore, targeting FSH-FoxO1 signaling may be beneficial in clinical therapy for follicular atresia-related disorders such as polycystic ovarian syndrome and premature ovarian failure.

## Materials and Methods

### Animals

All the animal experiments were performed in accordance with the guidelines of the Animal Research Institute Committee at Nanjing Agricultural University. Three-to-four-week-old female ICR mice (Qing Long Shan Co., Animal Breeding Center, Nanjing, China) were housed five per cage in a temperature-controlled (22±2 °C) room with a 12 : 12 h light:dark cycle (lights on from 07 00 to 1900 hours) and had *ad libitum* access to water and food. FSH administration was performed as previously described (also see Supplementary Figure S1).^32^ Briefly, to induce growth of DFs, mice were i.p. injected with FSH (Ningbo Second Hormone Factory, Ningbo, China) twice daily (12-h intervals, 08 00 and 2000 hours) for 2 days at a dose of 10 IU on day 1 and 5 IU on day 2. FSH was then withdrawn for an additional 24 or 48 h to promote natural follicular atresia in DFs, or injected i.p. (10 IU per mouse) 6 h before MGC retrieval. At 48, 72 and 96 h after the first FSH injection, MGCs were isolated from DFs (>200 *μ*m) in the left ovaries for qRT-PCR, immunoblotting and caspase activity detection. The right ovaries were fixed with 4% paraformaldehyde and embedded in paraffin for subsequent TUNEL assay and H&E staining. In this study, unless otherwise specified, MGCs were equal to MGCs from DFs.

### Cell culture

For primary MGC culture, the procedures were performed as described.^4^ In brief, 3- to 4-week-old ICR mice were injected i.p. with 10 IU pregnant mare serum gonadotropin and killed 48 h later.^63^ Superovulated mouse ovaries were harvested and individually transferred into 35-mm Petri dishes containing PBS and then punctured with a syringe to release MGCs from DFs (>200 *μ*m in diameter) under a surgical dissecting microscope. The cell suspensions were plated in DMEM/F-12 (1 : 1) (Invitrogen, Shanghai, China) supplemented with 10% fetal bovine serum (FBS; Gibco, Grand Island, NY, USA) and 100 units/ml penicillin plus 100 *μ*g/ml streptomycin (Gibco). After incubation for 4 days at 37 °C in 5% CO_2_, MGCs were washed and cultured in the presence or absence of FSH (ProSpecbio, Ness-Ziona, Israel) for the indicated times. NIH 3T3 cells (mouse embryonic fibroblasts, Institute of Biochemistry and Cell Biology, CAS, Shanghai, China) were grown in DMEM high-glucose medium (Invitrogen) with 10% FBS (Gibco) following previously reported protocols.^64^

### Cell treatments and transfection

#### FSH treatment

H89, LY294002, SB203580 and U0126 were purchased from Beyotime (Beijing, China). After exposure to 200 *μ*M H_2_O_2_ (Sigma, St. Louis, MO, USA) for 1 h, MGCs were rinsed with PBS and grown in serum-free DMEM/F-12 containing 7.5 IU/ml FSH for 2, 6, 12, 24 or 36 h. In some experiments, H89 (10 *μ*M), LY294002 (20 *μ*M), SB203580 (20 *μ*M) or U0126 (3 *μ*M) was added 30 min before FSH treatment.

#### Transfection

The FoxO1 expression vectors pcDNA3-FLAG-FKHR (WT), pcDNA3-FLAG-FKHR (AAA) and pcDNA3-FLAG-FKHR (F) were kindly provided by Dr. Haojie Huang (University of Minnesota, Minneapolis, MN, USA). pGL3-Basic and pRL-TK were purchased from Promega (Madison, WI, USA). pcDNA3 was obtained from Invitrogen. pGL3-FoxO1 (W) and pGL3-FoxO1 (M) were constructed in our lab. Transfection was performed using Lipofectamine 2000 Reagent (Invitrogen) according to the manufacturer's directions.

### Intracellular ROS detection

ROS levels were determined by measuring the oxidative conversion of dihydroethidium bromide to ethidium bromide, which generates red fluorescence when bound to DNA within the cell nuclei. The experiments were conducted using the GENMED Intracellular ROS Red Fluorescence Determination Kit (GENMED, Shanghai, China) according to the manufacturer's directions. Fluorescent images were obtained using a Carl Zeiss laser-scanning confocal microscope (Carl Zeiss, Jena, Germany). The optical density was evaluated in each MGC with ImageJ 1.42q software (National Institutes of Health, Bethesda, MD, USA).

### Measurement of caspase activity

Activities of caspases were measured as described previously.^65^ Caspase-3 activity was analyzed using the Caspase-3 Activity Assay Kit (Beyotime) with the fluorogenic substrate Ac-DEVD-pNA (acetyl-Asp-Glu-Val-Asp p-nitroanilide). Caspase-8 and -9 activities were measured using Ac-IETD-pNA (acetyl-Ile-Glu-Thr-Asp p-nitroanilide, Beyotime) and Ac-LEHD-pNA (acetyl-Leu-Glu-His-Asp p-nitroanilide, Beyotime), respectively. The experimental procedures were performed according to the manufacturer's instructions. Briefly, cells were digested in 0.25% Trypsin-EDTA (Gibco), pelleted by centrifugation at 3000 r.p.m. for 5 min, and lysed in 50 *μ*l lysis buffer for 15 min on ice. Whole-cell lysates were centrifuged at 20 000 × *g* for 15 min at 4 °C, and the supernatants were collected. The protein concentration was determined with the Bradford Protein Assay Kit (Beyotime). Ten micrograms of total protein was added to a 96-well plate containing 100 *μ*l buffer plus caspase substrates and incubated for 2 h at 37 °C. Cleavage of the substrates yielded a yellow product, p-nitroaniline, which was detected at 405 nm using a microplate reader (Bio-Rad, Hercules, CA, USA).

### Western blotting

Cells were harvested in RIPA lysis buffer, and protein was quantified with the BCA assay (Beyotime). Whole-cell lysates containing 15 *μ*g total protein were loaded onto 7.5% sodium dodecyl sulfate (SDS)-polyacrylamide gels, separated by electrophoresis, and transferred to polyvinylidene difluoride membranes (Millipore, Billerica, MA, USA). Nonspecific binding on the membranes was blocked with 5% bovine serum albumin (BSA) in TBS-T (50 mM Tris-HCl, pH 7.5, 150 mM NaCl, 0.1% Tween 20) for 1 h at room temperature. Membranes were then probed with primary antibodies (1 : 1000) against mouse FoxO1, DYKDDDDK Tag (Cell Signaling Technology, Danvers, MA, USA) and *α*-tubulin (Sigma) in blocking solution overnight at 4 °C. After washing with TBS-T, membranes were incubated with horseradish peroxidase-conjugated secondary antibodies (Santa Cruz Biotechnology, Santa Cruz, CA, USA) for 1 h at room temperature. Protein bands were visualized by exposing to an enhanced chemiluminescence detection system (LAS-4000 imager, Fujifilm, Tokyo, Japan) using the SuperSignal West Pico chemiluminescent substrate (Pierce, Rockford, IL, USA). Densitometry analyses were performed using ImageJ 1.42q software (National Institutes of Health), and the values for target proteins were normalized to *α*-tubulin as the endogenous control.

### qRT-PCR

Total RNA was isolated with TRIZOL reagent (Invitrogen) and reverse-transcribed into cDNA using the Reverse Transcription System (Promega) according to the manufacturer's instructions. Real-time PCR was performed with SYBR Premix Ex Taq (TaKaRa Biotechnology (Dalian) Co., Ltd., Otsu, Shiga, Japan) and gene-specific primers (see Supplementary Table S1 for primer sequences) on the 7300 Standard Real-Time PCR System (Applied Biosystems, Foster City, CA, USA). Melting curves were analyzed to verify amplification specificity. Expression data were normalized to the amount of *β*-actin expressed.

### Immunofluorescence experiments

MGCs were seeded on coverslips in 12-well plates. Cells were grown to 90% confluency in 4 days and then exposed to the treatments described above. After washing in PBS, the cell climbing sheets were fixed with 4% paraformaldehyde for 1 h, permeabilized using 0.5% Triton X-100 in PBS for 10 min at 4 °C and blocked with 1% BSA for 1 h at room temperature. The coverslips were then incubated with anti-FoxO1 antibody (Cell Signaling Technology; 1 : 100) for 1 h at 37 °C. The cells were incubated with Alexa Fluor 488 (green)-conjugated goat anti-rabbit IgG (Invitrogen; 1 : 200) for 1 h in the dark. After nuclear staining with DAPI (Invitrogen) for 20 min, the coverslips were washed, mounted on slides and observed under a laser-scanning confocal microscope (Carl Zeiss).

### H&E staining

H&E staining was performed as previously described.^66^ Briefly, at 48, 72 and 96 h after the first FSH injection, ovaries collected from mice primed with or without FSH were fixed with 4% paraformaldehyde, paraffin-embedded, serially sectioned at 5 *μ*m thickness and mounted on glass slides. After deparaffinization and rehydration, sections were stained with H&E (Nanjing Jianchen Institute of Biological Engineering, Nanjing, China) and examined using dot Slide-digital virtual microscopy (Olympus, Tokyo, Japan).

### TUNEL assay

Apoptosis was determined with the TUNEL method as described.^4^ The procedures were performed using the *In Situ* Cell Death Detection Kit (Roche Applied Science, Mannheim, Germany) according to the manufacturer's instructions. After TUNEL reactions, cell climbing sheets or ovarian sections were mounted with VECTASHIELD Mounting Medium plus DAPI and examined under a laser-scanning confocal microscope (Carl Zeiss).

### PKA assay

The enzymatic activities were detected using a GENMED Cellular PKA Activity Colorimetric Quantitative Determination Kit, a GENMED Cellular PI3K Activity Colorimetric Quantitative Determination Kit and a GENMED Cellular PKB/AKT Activity Colorimetric Quantitative Determination Kit (GENMED) according to the manufacturer's instructions. In brief, cells were cultured in T-75 flasks, removed carefully with a cell scraper (Corning Incorporated Life Sciences, Acton, MA, USA), pelleted by centrifugation at 3000 r.p.m. for 5 min, and lysed with 500 *μ*l lysis buffer for 30 min on ice. Cell lysates were then centrifuged at 20 000 × *g* for 20 min at 4 °C. Total proteins in the supernatants were quantified with the BCA reagent (Beyotime). Protein (50 *μ*g in 5 *μ*l) was transferred to a 96-well plate in which each well contained 95 *μ*l reaction mixture and incubated for 5 min at 37 °C. Samples were then read with a microplate reader (Bio-Rad).

### Luciferase reporter gene assay

The *FoxO1* promoter was amplified with PCR from mouse genomic DNA (bases 78–2125, 5′-UTR of *FoxO1*) containing a FRE (AGTAAACAAA), cloned into the pGL3-Basic plasmid (Promega) at the *Kpn*I and *BgI*II (TaKaRa Biotechnology (Dalian) Co., Ltd.) sites and named pGL3-FoxO1 (W) (see Supplementary Figure S3 for primer sequences). pGL3-FoxO1 (M) was obtained by introducing four mutations into FRE using a Mut Express II Fast Mutagenesis Kit (Vazyme, China). Mouse NIH 3T3 fibroblasts were seeded in a 12-well plate and grown to 70% density before transfection with Lipofectamine 2000 (Invitrogen). Three plasmids were co-transfected in each treatment: (1) 1.6 *μ*g expression vector pcDNA3-FLAG-FKHR (WT); (2) 1.6 *μ*g reporter construct pGL3-FoxO1 (W) or pGL3-FoxO1 (M); and (3) 32 ng control reporter pRL-TK (Promega). After 36 h, cells were processed for reporter assays using the Dual-luciferase Reporter Assay System (Promega) and Modulus Microplate Luminometer (Turner BioSystems, Sunnyvale, CA, USA) according to the manufacturer's instructions. All inserts were verified with sequencing.

### ChIP assays

ChIP assays were performed according to the manufacturer's protocols in the Pierce Agarose ChIP Kit (Pierce). Briefly, cells were fixed in 1% formaldehyde for 10 min at room temperature. Crosslinking was stopped by adding glycine to a final concentration of 125 mM. After washing with cold PBS containing protease inhibitors, cells were lysed in 1 ml SDS lysis buffer (1% SDS, 10 mM EDTA, 50 mM Tris, pH 8.1). Chromatin was then sheared with sonication to generate fragments 250–750 bp. For each ChIP reaction, 10% of the chromatin was stored as input, and the rest was processed for immunoprecipitation by incubating with anti-FoxO1 (Cell Signaling Technology) and rabbit IgG (as control). Proteinase K was used to degrade proteins in the precipitated complexes, and DNA was isolated and used as a template for qRT-PCR using the primers indicated in Supplementary Table S1. qRT-PCR products were then electrophoresed on a 2% agarose gel. The amount of immunoprecipitated DNA for each ChIP reaction is presented as the percent of input chromatin.

### Statistical analysis

Data were analyzed using SPSS version 16.0 (SPSS Inc., Chicago, IL, USA). Statistical significance was calculated with the Student's *t*-test (*P*<0.05 was considered statistically significant). All experiments were repeated at least three times. Results were expressed as the mean±S.E.

## Abbreviations

AKT: protein kinase B
BSA: bovine serum albumin
ChIP: Chromatin immunoprecipitation
DFs: dominant follicles
DBD: DNA-binding domain
ERK1/2: extracellular signal-regulated kinases 1 and 2
ER: estradiol receptor
FSH: follicle-stimulating hormone
FRE: FoxO-recognized element
FasL: Fas ligand
H&E: hematoxylin and eosin
i.p.: intraperitoneal
LH: leutinizing hormone
MGCs: mouse granulosa cells
PBS: phosphate-buffered saline
PI3K: phosphatidylinositol-3 kinase
p38-MAPK: p38 mitogen-activated protein kinases
PKA: protein kinase A
qRT-PCR: quantitative reverse transcription polymerase chain reaction
ROS: reactive oxygen species
SDS: sodium dodecyl sulfate
TBS-T: 50 mM Tris-HCl, pH 7.5, 150 mM NaCl, 0.1% Tween 20
TRAIL: tumor necrosis factor-related apoptosis-inducing ligand
TUNEL: terminal deoxynucleotidyl transferase-mediated dUTP nick end labeling
WT: wild-type
