# Supplementary figures and images for: Involvement of FoxO1 in the effects of follicle-stimulating hormone on inhibition of apoptosis in mouse granulosa cells
Source: Cell Death Dis. 2014 Oct 16;5(10):e1475–. doi: 10.1038/cddis.2014.400 (PMC4237239; doi:10.1038/cddis.2014.400)

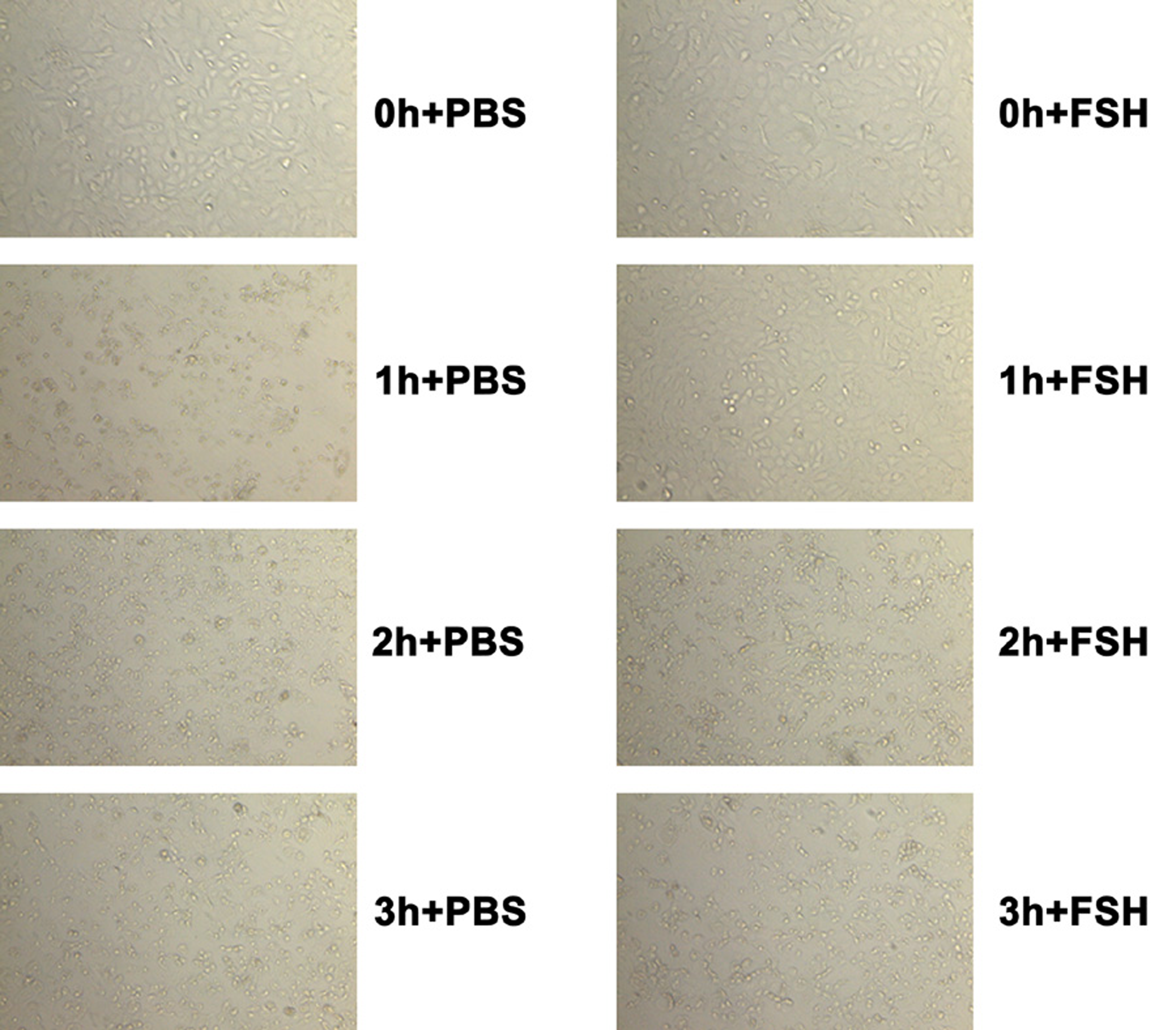

Supplement: Supplementary Figure S2 [file cddis2014400x2.tif]
