# Supplementary material for: Involvement of FoxO1 in the effects of follicle-stimulating hormone on inhibition of apoptosis in mouse granulosa cells
Source: Cell Death Dis. 2014 Oct 16;5(10):e1475–. doi: 10.1038/cddis.2014.400 (PMC4237239; doi:10.1038/cddis.2014.400)
Supplement: Supplementary Figure Legends [file cddis2014400x7.doc]

# Supplementary Files

# Supplementary Figure S1 The mouse ovarian stimulation procedure. (A-E) To induce growth of DFs in mouse ovaries under physiological conditions, we injected mice i.p. with FSH twice daily (12-h intervals) for 2 days at a dose of 10 IU on day 1 and 5 IU on day 2 (A). FSH was then withdrawn for an additional 24 or 48 h to promote natural follicular atresia in DFs (B, D), or injected i.p. (10 IU/mouse) 6 h before MGC collection (C, E). At 48, 72, and 96 h after the first FSH injection, mouse ovaries or MGCs of DFs were harvested for tests.

# Supplementary Figure S2 MGCs maintained a normal appearance upon FSH stimulation after 1 h of H_2_O_2_ exposure. MGCs collected from DFs were incubated with 200 μM H_2_O_2_ for 0 (vehicle), 1, 2, or 3 h, washed with PBS, and cultured in serum-free medium containing 7.5 IU/ml FSH for 12 h. Images were obtained using an inverted phase contrast microscope. Magnification was 200×.

**Supplementary Figure S3** Overview of FoxO1 reporter constructs. (A) The 2468-bp 5'-UTR sequence of *FoxO1*. Red, translation initiation site. Yellow, transcription start site. Blue, FRE. The region between the pink DNA was amplified with PCR and cloned into the pGL3-Basic plasmid. (B) Map of the pGL3-FoxO1 luciferase reporter vector. The red rectangles showed the restriction endonucleases used for cloning. (C) *FoxO1* promoter amplification primers. Cyan, protective bases. Red, restriction enzyme cutting site. Pink, primer sequences.

**Supplementary Figure S4** Identification of FoxO1 upstream regulators in response to FSH signalling. Primary cultured MGCs were exposed to 200 μM H_2_O_2_ for 1 h and then grown in serum-free medium containing 7.5 IU/ml FSH for 6 h. H89 (10 μM) was added 30 min before FSH treatment. The effects of the FSH signalling inhibitors on gene expression were determined with qRT-PCR. Expression data were normalized to that of β-actin. Pearson correlation coefficients were analysed using SPSS version 16.0 to examine the relationship between the expression patterns of *FoxO1* and the candidate genes. The data represent the means ± S.E. (n = 3).

**Supplementary Figure S5** Schematic model of FoxO1-dependent apoptosis regulated by FSH in MGCs from DFs. Under stress caused by FSH deprivation or ROS, *FoxO1* is activated and induces the expression of multiple target genes, including *FoxO1* itself and pro-apoptotic genes, which in turn transmit death signals through the mitochondrial pathway, finally leading to caspase-3 activation, MGC apoptosis, and follicular atresia. However, in the presence of FSH, the PKA-PI3K-AKT axis blocks the nuclear translocation of FoxO1, terminating the positive feedback regulation of *FoxO1* transcription and target gene expression, thus preventing FoxO1-dependent apoptosis in DFs.

**Supplementary Figure S6** Chromatin in MGCs was sheared with sonication to generate fragments 250-750 bp. Columns 1 and 2 represent two repeated electrophoretic results of the sonicated DNA sample.
