# Supplementary material for: Involvement of FoxO1 in the effects of follicle-stimulating hormone on inhibition of apoptosis in mouse granulosa cells
Source: Cell Death Dis. 2014 Oct 16;5(10):e1475–. doi: 10.1038/cddis.2014.400 (PMC4237239; doi:10.1038/cddis.2014.400)
Supplement: Supplementary Table S1 [file cddis2014400x8.doc]

**Supplementary Table S1 Primer sequences**

| **Gene Name** | **GenBank Accession NO.** | **Primer Sequence(5'→3')** |
| --- | --- | --- |
| *FoxO1* | [GenBank: NM_019739.3] | F: CGTGCTTACAGCCTTCTA  R: ACCTCCATCGTGACAAAA |
| *FoxO3* | [GenBank: NM_019740.2] | F: AGCCGTGTACTGTGGAGCTT  R: TCTTGGCGGTATATGGGAAG |
| *FoxO4* | [GenBank: NM_018789.2] | F: TCATCAAGGTTCACAACGAGGC  R: AGGACAGACGGCTTCTTCTTGG |
| *Bim* | [GenBank: NM_207681.2] | F: TATGGAGAAGGCATTGAC  R: TGTGGTGATGAACAGAGG |
| *β-actin* | [GenBank: NM_007393.3] | F: GCTGTCCCTGTATGCCTCT  R: GTCTTTACGGATGTCAACG |
| *ERalpha* | [GenBank: NM_007956.4] | F: GGTCAGTGCCTTGTTGGATGCT  R: ACGGTGGATGTGGTCCTTCTCTT |
| *Chx-10* | [GenBank: NM_007701.2] | F: CCCAAACCTGTCTCACG  R: AGGGCTCACCAGCAGTA |
| *Evi-1-Isoform-1* | [GenBank: NM_007963.2] | F: GCGAACCTAACACGGCACTTGA  R: CCTCCTCATCCAACAACACCTCATC |
| *C/EBPbeta* | [GenBank: NM_009883.4] | F: GGACAAGCTGAGCGACGAGTA  R: ACCTTGTGCTGCGTCTCCA |
| *Pou3f1* | [GenBank: NM_011141.2] | F: CAGTTCAAGCAACGACGCATCA  R: TCGGTCTCCTCCAGCCACTT |
| *RORalpha* | [GenBank: NM_013646.2] | F: TCAGCAGAGCAATGCCACCTA  R: CATCCGACCAAACTTGACAGC |
| *Pax-5* | [GenBank: NM_008782.2] | F: GACATCTTCACCACCACGGAAC  R: GGTTGTGCTCGCCAAGTCTC |
| *PPARgamma* | [GenBank: NM_001127330.1] | F: CGTGAAGCCCATCGAGGACA  R: TGGAGCACCTTGGCGAACAG |
| *FoxO1* (Primer for ChIP) | [GenBank: NC_000069.6] | F: CGGAGCGGGCTTGAGTGGAA  R: TAGGAGGCAAACCGGCGTGGAG |
